# Clozapine Use During Pregnancy and Lactation: A Case-Series Report

**DOI:** 10.3389/fphar.2018.00264

**Published:** 2018-03-27

**Authors:** M. Luisa Imaz, Giovanni Oriolo, Mercè Torra, Dolors Soy, Lluïsa García-Esteve, Rocio Martin-Santos

**Affiliations:** ^1^Perinatal Psychiatry Program, Department of Psychiatry and Psychology, Hospital Clínic, Institut d'Investigacions Biomèdiques August Pi i Sunyer (IDIBAPS), Barcelona, Spain; ^2^Department of Medicine, University of Barcelona, Barcelona, Spain; ^3^Department of Psychiatry and Psychology, Hospital Clínic, Institut d'Investigacions Biomèdiques August Pi i Sunyer (IDIBAPS), Barcelona, Spain; ^4^Pharmacology and Toxicology Laboratory, Biochemistry and Molecular Genetics Service, Biomedical Diagnostic Center (CBD), Hospital Clínic, Institut d'Investigacions Biomèdiques August Pi i Sunyer (IDIBAPS), Barcelona, Spain; ^5^Division of Medicines, Pharmacy Service, Hospital Clínic, Institut d'Investigacions Biomèdiques August Pi i Sunyer (IDIBAPS), University of Barcelona, Barcelona, Spain; ^6^Department of Psychiatry and Psychology, Hospital Clinic, Institut d'Investigacions Biomèdiques Artur Pi i Sunyer (IDIBAPS), Centro de Investigación Biomédica en Red en Salud Mental (CIBERSAM), Barcelona, Spain

**Keywords:** clozapine, pregnancy, placental, pharmacokinetics, amniotic, lactation, neonatal, schizophrenia

## Abstract

The current prescription of clozapine in psychotic women of reproductive age makes it crucial to understand its pharmacokinetics during pregnancy and lactation as well as its risk profile for neonatal outcome. The aim of this case series was to provide new evidence on the pharmacokinetic features of clozapine that determine its passage through the placenta and amniotic fluid, as well as the neonatal clozapine elimination half-life (t1/2). This case series demonstrates for the first time that clozapine might show partial placental passage similar to other atypical antipsychotics. Clozapine levels decreased during the first few days in nursing infants. The half-life of clozapine in neonates was slightly higher than previously estimated. Clozapine use in pregnancy may be associated with diabetes mellitus, especially if there is a family history of this disease. Although no acute toxicological effects were observed in the intrauterine exposed newborn, close follow-up of pregnancy is recommended. However, these results must be taken with caution being a case series with small sample size

## Introduction

Clozapine is a second-generation antipsychotic agent, chemically a di-benzodiazepine, that is approved for treatment-resistant schizophrenia and risk reduction of recurrent suicidal behavior in schizophrenia or schizoaffective disorder. It is also used off-label to reduce craving in patients with substance use disorder (SUD) comorbidity (Hasan et al., 2015). Clozapine acts as an antagonist of serotonin 5HT2, dopamine D2, α1-adrenergic, muscarinic and H1 histaminic receptors and it induces relatively few extrapyramidal effects (Stahl, 2017). Unlike some first- and second-generation antipsychotics, it does not cause sustained increases in serum prolactin levels and does not affect other hormonal levels sufficiently to inhibit fertility (Haddad and Wieck, 2004).

A recent systematic review of the safety of clozapine during pregnancy and lactation found very limited data on the topic (Mehta and Van Lieshout, 2017). In pregnant women, clozapine appears to be associated with an increased risk of gestational diabetes mellitus compared to mothers not taking any antipsychotic (Bodén et al., 2012), as also observed in several case reports (Waldman and Safferman, 1993; Dickson and Hogg, 1998; Nguyen and Lalonde, 2003; Karakula et al., 2004). Regarding the consequences for fetuses exposed to clozapine during pregnancy, a summary paper showed five cases of congenital malformations in 61 children (8.2%) (Dev and Krupp, 1995). A Novartis follow-up identified 22 fetal congenital malformations in 523 (4.2%) mothers treated with clozapine (McKenna et al., 2005). In a cohort study, women taking clozapine and/or olanzapine showed an increased risk for macrocephaly (Bodén et al., 2012). Moreover, a case series reported seven spontaneous abortions in 84 women exposed to clozapine (Bazire, 2005). Some case reports have described shoulder dystocia (Waldman and Safferman, 1993; Dickson and Hogg, 1998), atrial septum defect (Vavrusova and Konikova, 1998), and ectopic anus (Reis and Källén, 2008). Regarding the risk to infants, floppy infant syndrome (Di Michele et al., 1996) and a decrease in heart rate variability (Yogev et al., 2002; Coston et al., 2012; Guyon et al., 2015), seizure (Stoner et al., 1997), gastroesophageal problems, and delayed peristalsis (Karakula et al., 2004) have been observed. Concerning the clinical outcome associated with clozapine exposure during breastfeeding, data are even more scarce. One study reported delayed speech acquisition in an infant exposed to clozapine during 1 year of lactation (Mendhekar, 2007); an episode of agranulocytosis and a case of drowsiness were observed in another four cases (Dev and Krupp, 1995). Five cases of *in-utero* and lactation exposure to clozapine reported fewer sleep disturbances compared to those not exposed (Shao et al., 2015). Finally, there are few data on the long-term outcome of children exposed to clozapine during pregnancy. A case-control study showed a lower adaptive behavior (Bayley-III) score in children exposed to clozapine vs. other atypical antipsychotics (Shao et al., 2015). Lastly, a case report observed the retardation of psychomotor development in a child after 6 years of follow-up (Tenyi and Trixler, 1998).

The heterogeneity of studies and clinical outcomes based on distinct clozapine daily doses, different timing of exposures during pregnancy and lactation, and the usage of concomitant drugs in some cases leaves many questions unanswered (Mehta and Van Lieshout, 2017). It has been suggested that the accumulation of clozapine in fetal serum may be associated with an increased rate of neonatal complications (Dev and Krupp, 1995). There are currently two cases in the literature reporting neonatal clozapine blood concentrations. In the first, Barnas et al. (1994) suggested transplacental passage and relative accumulation of clozapine in the neonate. The same trend for clozapine accumulation was observed in breastmilk. The second case (Moreno-Bruna et al., 2012) described a newborn with delayed peristalsis on the second day of life, with an increase in half-life elimination of the drug suggesting a substantial newborn plasma clozapine concentration on the fifth and eighth day of life.

Given the current increase in the prescription of atypical antipsychotics, such as clozapine, in young psychotic women (Toh et al., 2013), it seems important to know better their pharmacokinetics and adverse effects during pregnancy and lactation as well as its risk profile for neonatal outcome.

## Methods

We report four cases of three Caucasian women who attended the outpatient Perinatal Psychiatry Program of a teaching hospital during pregnancy and postpartum with clozapine. Complementary to their obstetric and psychiatric care, clozapine and norclozapine plasma concentrations were determined in the mother–infant pair on the day of delivery [intrapartum maternal blood (IMB), fetal umbilical cord blood (UCB), and amniotic fluid (AF)] to calculate the infant–maternal plasma clozapine concentration ratio. The neonatal plasma concentration of clozapine was measured several times after delivery to observe half-life elimination in those neonates with artificial lactation (Food and Drug Administration, 2005). Only in one case it was possible to study the plasma concentrations of clozapine in both the mother and the neonate at 33 h postpartum to calculate the lactation transfer. Obstetric and pediatric outcomes were reviewed from medical records. Pharmacological treatment was administered with the consent of the patients. All patients gave written informed consent for the presentation of their data.

The drug was analyzed in venous blood samples drawn in the morning before a first daily dose of clozapine, and under steady-state conditions. The blood samples were allowed to clot at room temperature for 15–30 min, and then centrifuged at 1800 × g/10 min. Plasma was stored at −20°C until analysis. Plasma concentrations of clozapine/norclozapine, were measured using a validated high-performance liquid chromatography method, with UV diode array detection and solid- phase extraction (Gupta, 1995; Liu et al., 2001). For amniotic fluid, drugs were extracted using a double-step liquid–liquid procedure (Titier et al., 2003). The within- and between-day precision expressed as the coefficient of variation (CV) % were both <10%. The limit of quantification (LOQ) was 5 ng/mL. Assuming linear pharmacokinetic behavior, the elimination half-life (t_1/2_) was calculated by means of 0.693/ke. The elimination rate constant (ke) was determined from the neonate venous blood clozapine concentration data vs. time after birth by linear least squares regression analysis of the terminal portion of the log concentration-time curve. Drug urine screening and cotinine quantification were performed by immunoassay.

The aim of the present case-report series was to provide new information on the features of clozapine pharmacokinetics that determine its placental and lactation passage, as well as the neonatal clozapine elimination half-life (t_1/2_) and neonatal and infant/child outcomes.

### Case series

**Figure 1:**
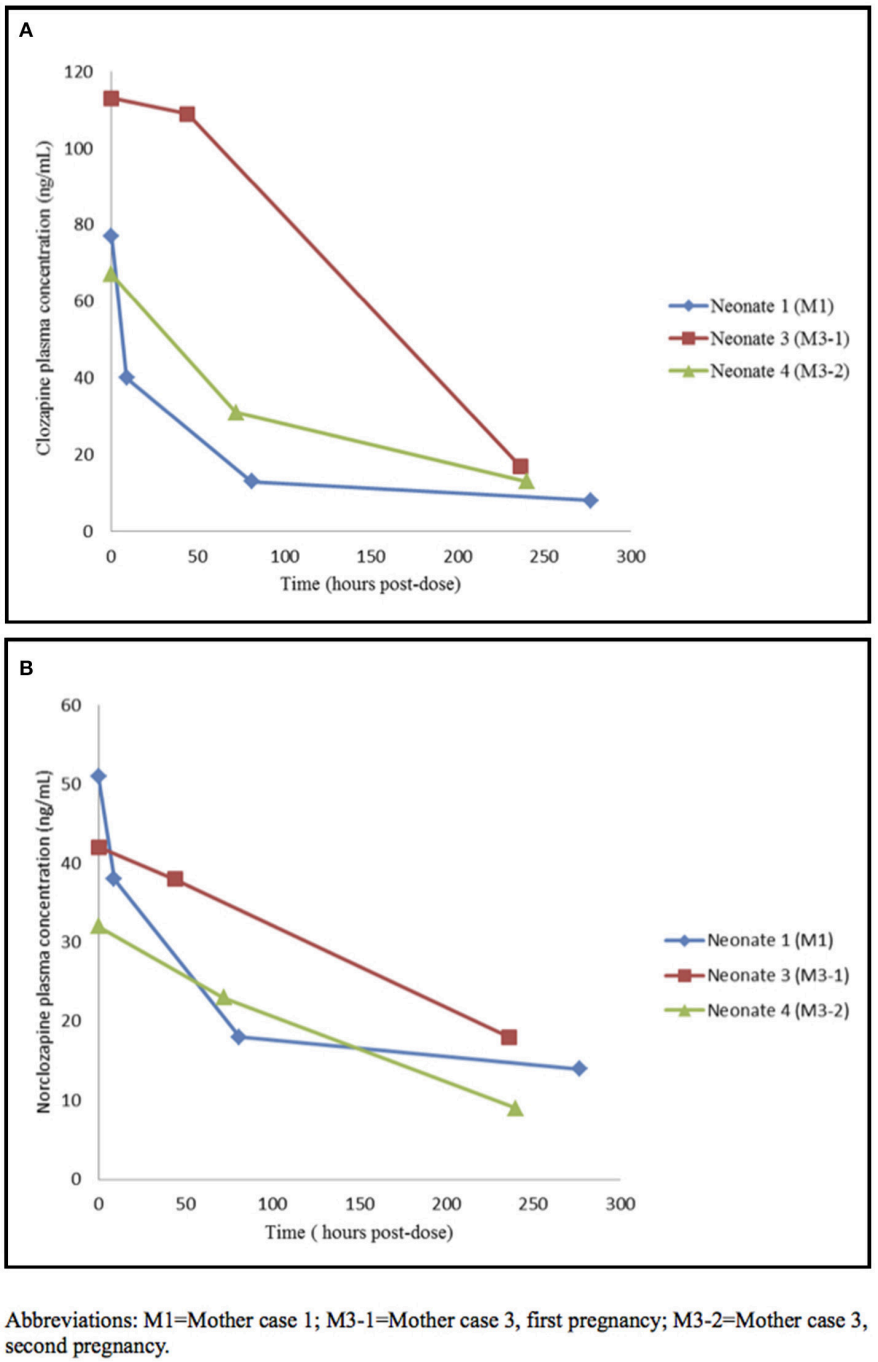
Neonatal clozapine **(A)** and norclozapnie **(B)** plasma concentration time profile. M1, Mother case 1; M3-1, Mother case 3, first pregnancy; M3-2, Mother case 3, second pregnancy.

The first patient (M1) was a 37-year-old woman with paranoid schizophrenia since the age of 24. She was treated with risperidone 5 mg/day. Due to an episode of amenorrhea with galactorrhea, risperidone was replaced by quetiapine 300 mg/day. At 34 years of age, she required hospital admission for a new psychotic episode (Table 1). Clozapine was initiated and titrated up to 550 mg/day with partial remission. Thirty sessions of ECT were administered, and long-acting intramuscular (LAI) risperidone was added (50 mg/14 days). After 3 years in stable remission, she expressed the wish to have a child and her intrauterine device (IUD) was removed. She was a primipara, with a body mass index of 31.84, and no other medical problems. When pregnancy was confirmed she was taking clozapine 550 mg/day and LAI risperidone 50 mg/month and she was smoking 18 cigarettes/day. She subsequently reduced the number of cigarettes/day by 50%, and clozapine was progressively titrated down to 350 mg/day; the LAI risperidone dose was maintained. No agranulocytosis was detected. Four ultrasounds during gestation showed physiological fetal well-being parameters (Table 1). A spontaneous vaginal delivery was carried out in the 38th week of pregnancy, with no perinatal complications. No agranulocytosis, seizures, or other neonatal complications were observed (Table 2). The plasma concentrations of clozapine/ norclozapine in IMB, UCB, and AF ratios are displayed in Table 3. Figure 1 shows the half-life neonatal elimination of clozapine. Artificial lactation was carried out. No psychotic relapse was recorded. In the follow-up of the child at 6 years of age, symptoms of attention deficits and hyperactivity were present without any diagnostic criteria for ADHD.

**Table 1:** Maternal characteristics and pregnancy-related outcomes in the case series and in the previous cases reported in the literature.

**Variables**	**Chronic clozapine exposure**						**Acute clozapine exposure**	
	**Mother 1 (M1) 2007**	**Mother 2 (M2) 2014**	**Mother 3 (M3-1) 2015**	**Mother 3 (M3-2) 2016**	**Barnas et al., 1994**	**Moreno-Bruna et al., 2012**	**Kłys et al., 2007**	**Novikova et al., 2009**
**MATERNAL VARIABLES**								
Psychiatric diagnosis	CLZ- R Schizophrenia	Schizoaffective	Schizoaffective		Schizophrenia	Schizophrenia	Schizophrenia	Self-intoxication
Concurrent diagnosis	No	No	SUD		No	No	Self-intoxication	No
Age (years)^*^	37	34	33	34	31	27	34	16
Ethnicity	Caucasian	Caucasian	Caucasian		NA	NA	NA	NA
Educational level	High School	High School	High School		NA	NA	NA	NA
Marital status	Married	Married	Living together		NA	NA	Married	NA
Employment status	Employed	Employed	Unemployed		NA	NA	NA	NA
Planned pregnancy	Planned & happy	Planned & happy	Unplanned & happy		Planned	Planned	NA	NA
Parity	Primiparous	Primiparous	Primiparous	Multiparous	NA	Multiparous	NA	NA
BMI (pre-pregnancy)—Kg/m^2^	31.84	27.78	24.90	28.09	NA	NA	25.00	NA
Pregestational diabetes	No	No	No	No	NA	NA	No	NA
Family history of diabetes	No	Yes	No	No	NA	NA	NA	NA
**PREGNANCY VARIABLES**								
Weight gain in pregnancy—Kg	13	16	11.5	11.7	25	NA	16.00	NA
Gestational diabetes	No	Yes at 14 gwk	No	No	NA	No	No	No
Other complications	No	No	IUGR-1 at 28 g wk	No	NA	No	No	No
Type of delivery	Vaginal	Cesarean^†^	Cesarean^‡^	Cesarean^¶^	Vaginal^**^	Vaginal	Vaginal	Cesarean^†^
Maternal duration of gestation^††^	38+6	40+5	38+6	39	41+0	39+0	40+0	32+0

*BMI: body mass index; CLZ-R Schizophrenia: clozapine-resistant schizophrenia; GWK: gestational week; IUGR: intrauterine growth retardation; SUD: substance use disorder*.

* Age at the time of pregnancy;

† Emergency cesarean section;

‡ Elective cesarean section due to breech presentation;

¶ Elective cesarean section for short interpregnancy period;

** Instrumental vaginal;

†† *Maternal duration of gestation: weeks + days after last menstrual date*.

**Table 2:** Neonatal, infant, and child outcomes in the case series and in the previous cases reported in the literature.

**Variables**	**Chronic clozapine exposure**						**Acute clozapine exposure**	
	**Neonate 1 (M1) 2007**	**Neonate 2 (M2) 2014**	**Neonate 3 (M3-1) 2015**	**Neonate 4 (M3-2) 2016**	**Barnas et al., 1994**	**Moreno-Bruna et al., 2012**	**Kłys et al., 2007**	**Novikova et al., 2009**
**NEONATAL OUTCOMES**								
Infant sex	Male	Female	Male	Female	Female	Male	Male	Female
Estimated gestational age	38+6	40+5	38+6	39	41+0	39+0	40+0	32+0
Apgar score (1/5/10 min)	9/10/10	9/10/10	6/10/10	9/10/10	5/8/–	1/–/–	10/10/–	–/5/–
Arterial cord pH	7.09	7.18	7.21	N/A	7.34	NA	N/A	7.19
Infant weight (g) (%tile)	3,850 (p75-80)	3,660 (p85-90)	2,498 (*p* < 3)	3,650 (p85-9)	3,600 (p80-85)	4,050 (*p* > 95)	4,060 (p90-95)	1,700 (p23)
Length (cm) (%tile)	51.50 (p90-95)	51.00 (p80-85)	47.00 (p10)	49.00 (p50)	N/A	NA	55.50 (*p* > 95)	NA
Cranial perimeter (cm) (%tile)	36.00 (p85-90)	36.00 (*p* > 95)	34.00 (p25)	35.50 (p90-95)	N/A	NA	36.50 (p90-95)	NA
NICU admission	No	No	Yes	No	No	NA	No	NA
Hospital stay (days)	4	3	4	4	N/A	20 min	17	NA
Congenital anomaly	No	No	Inguinal hernia and cryptorchi dism (left)	No	No	No	No	No
Cardiovascular	No	No	No	No	No	No	No	Abnormal fetal CTG
Respiratory	No	No	Resuscitation procedures^*^	No	No	Resuscitation procedures^*^	No	
Others^†^	No	No	No	No	No	No	Delayed peristalsis	Delayed peristalsis
Blood leukocytes UCB (x 10^9^/L)	25.17	22.23	8.65^‡^	12.49	NA	NA	Normal	NA
Urine drug test^¶^ (1st day PP)	NA	NA	BZD (+)	NA	NA	Etanol (-)	NA	NA
Infant feeding	Formula	Mixed BF 5 days, then formula	Formula	Formula	Formula	NA	Formula	NA
**INFANT/CHILD OUTCOMES**								
Developmental abnormalities	TDH symptom at 6 years	No	General neurodeve-lopmental delay	No	No	NA	No	No
Last observation	10 years	32 months	16 months	5 months	6 months	20 min	2 years	6 days

*BZD, benzodiazepines; BF, breastfeeding; CGT, cardiotocography tracing; NICU, neonatal intensive care unit; PP, postpartum; UCB, umbilical cord blood; g, gram; cm, centimeter; %tile, percentile*.

* Positive pressure ventilation;

† Others: renal, thyroid, gastrointestinal, central nervous system;

‡ At 2nd day postpartum;

¶ *Urine drug test included alcohol, cannabis, heroin, methadone, cocaine, benzodiazepines*.

**Table 3:** Placental passage and neonatal half-life values of clozapine and metabolite in the case series and in the previous reported in the literature.

**Variable**	**Chronic clozapine exposure**						**Acute clozapine exposure**	
	**Mother 1 (M1) 2007**	**Mother 2 (M2) 2014**	**Mother 3 (M3-1) 2015**	**Mother 3 (M3-2) 2016**	**Barnas et al., 1994**	**Moreno-Bruna et al., 2012**	**Kłys et al., 2007**	**Novikova et al., 2009**
**MATERNAL VARIABLES**								
Psychiatric diagnosis	CLZ-R Schizophrenia	Schizoaffective	Schizoaffective		Schizophrenia	Schizophrenia	Schizophrenia	No
Co-ocurrent diagnosis	No	No	SUD^*^		No	No	No	No
CLZ therapy duration	wk 0-delivery	wk 0-delivery	wk0-16, 21-delivery	wk 0-delivery	wk 0-delivery	wk 0-delivery	wk 0-12 self-intox wk39	Self-intox wk 32
Other medication during pregnancy	LA-RIS 50 mg/month wk 0-34	No	DZ 15 mg/day wk 30-37	SER 100 mg/day wk 35-delivery	No	No	VAL, PROM RIS, FX	No
Drug use during pregnancy	Tobacco daily	No	Alcohol, cocaine, cannabis^†^ Tobacco daily	Tobacco daily	NA	NA	No	NA
Urine drug test during pregnancy^‡^	(+) Cotinine	Negative	(+) Cotinine	(+) Cotinine	NA	NA	No	NA
Maternal CLZ dose at delivery (mg/day)	350	100	200	200	50	100	1000	2000
**[CLZ] AT DELIVERY (ng/ml)**							^¶^	^**^
Umbilical cord blood (UCB)	77	68	113	67	27	NA	NA	NA
Amniotic fluid (AF)	61	NA	67	NA	11.60	NA	NA	NA
Maternal plasma (MP)	198	122	194	148	14.10	NA	NA	NA
CLZ UCB/MP ratio (%)	38.88	55.70	58.24	45.27	191.48	NA	NA	NA
**[NORCLZ] AT DELIVERY (ng/ml)**								
Umbilical cord blood (UCB)	56	26	42	32	NA	NA	NA	NA
Amniotic fluid (AF)	39	NA	52	NA	NA	NA	NA	NA
Maternal plasma (MP)	200	79	114	149	NA	NA	NA	NA
NorCLZ UCB/MP ratio (%)	28.00	32.91	36.84	21.47	NA	NA	NA	NA
**NEONATAL HALF-LIFE (HOURS)**								
CLZ	99	NA	71.42	106.61	NA	NA	NA	NA
NorCLZ	161.16	NA	187.30	130.75	NA	NA	NA	NA

*CLZ, clozapine; CLZ-R, Schizophrenia, clozapine resistant schizophrenia; DZ, diazepam; FX, fluoxetine; LA-RIS, long acting risperidone; NA, not available; NorCLZ, norclozapine; PROM, prometazine; Self-intox, self intoxication; SER, sertraline: VAL, valproate; wk, week*.

* SUD, alcohol, nicotine, cannabis, or cocaine substance use disorder;

† first 5 month of pregnancy;

‡ Urine drug test included alcohol, cannabis, heroin, metadone, cocaine, benzodiazepines, and cotinine;

¶ Postmortem (at 24 h), Blood (μg/ml): [CLZ] 7.3, [NorCLZ] 2.3, [CLZ-N-oxide] 0.5. Liver (μg/ml): [CLZ] 28.0, [NorCLZ] 17.1, [CLZ-N-oxide] 31.1. Kidney (μg/ml): [CLZ] 10.1, [NorCLZ] 6.1, [CLZ-N-oxide] 5.8;

** *Qualitative urine analysis of CLZ in the mother: (+)3 days after CLZ intake, and in urine of the neonate: (+) 6 days after maternal CLZ intake*.

The second patient (M2), a 34-year-old woman, was diagnosed with schizoaffective disorder at the age of 18. She was treated initially with haloperidol 15 mg/day, and later on with sertindole 16 mg/day. Because of severe hypotension the treatment was changed to amisulpride 150 mg/day and risperidone 6 mg/day. She remained in remission for several years. At 30 years of age, she decided to plan a pregnancy. Due to an increase in prolactin level the risperidone dose was reduced to 4.5 mg/day. However, as the patient started being symptomatic, the 6 mg/day dose was reinstated and the patient became asymptomatic. After more than 3 years of follow-up without becoming pregnant, she switched treatment to 200 mg/day clozapine and after 6 months she became pregnant and she returned to our program (Table 1). In the 19th week of pregnancy, clozapine treatment was titrated down progressively to a dose of 100 mg/day to avoid fetal accumulation (Barnas et al., 1994). She developed diabetes during pregnancy that was successfully controlled with long-acting insulin at 6 U/day. Ultrasound examinations detected fetal macrosomia in the 29th week of gestation. No other physiological parameters were altered. She gave birth in the 40th week pregnancy by cesarean delivery. No perinatal complications were recorded and there was a good obstetric outcome (Table 2). No agranulocytosis, seizures or other neonatal complications were observed. Table 3 shows the plasma concentrations of clozapine/norclozapine in IMB and UCB. Mixed breastfeeding was conducted for 5 days. At 33 hours after delivery, the infant/mother clozapine ratio had decreased by 48.9%. However, 5 days after delivery, the mother was briefly hospitalized due to a relapse of manic psychotic symptoms, which responded rapidly to an increase in clozapine to 200 mg/day, and breastfeeding was stopped. No neurodevelopmental disorders were detected in the infant after 32 months follow-up.

The third case (M3-1), a 33-year-old woman, had a severe schizoaffective disorder comorbid with SUD for 8 years. She was treated with several antipsychotic treatments (risperidone 3 mg/day, LAI risperidone 50 mg/month, olanzapine 20 mg/day, paliperidone 9 mg/day, and aripiprazol 15 mg/day) without achieving clinical stability. Finally, treatment was switched to clozapine 200 mg daily. In 2014 she had one first trimester spontaneous abortion. In 2015, when she knew that she was pregnant again, she tried to stop antipsychotic medication at week 16, but the reappearance of auditory hallucinations forced her to reintroduce clozapine 200 mg daily at week 21. However, in week 26, due to psychotic decompensation, cocaine and alcohol abuse, as well as domestic abuse, she was hospitalized until the end of pregnancy and clozapine was increased up to 300 mg/day and diazepam 10 mg/day was added to achieve clinical remission. Ultrasound examinations showed type I intrauterine growth restriction in the 28th week of gestation. She gave birth to a boy in the 38th week of pregnancy by cesarean delivery due to breech presentation (Table 1). The Apgar-min 1 score was 6 and neonatal resuscitation was initiated in the delivery room. The Apgar-min 5 score was 10, and arterial cord PH was 7.21. It was also noted that the baby had a left inguinal hernia and left cryptorchidism. The urine drug test was positive for benzodiazepines. No agranulocytosis, seizures, or other neonatal complications were observed. Cerebral and cardiac ultrasounds were normal (Table 2). The plasma concentrations of clozapine/norclozapine in IMB, UCB, and AF were also measured (Table 3). Figure 1 shows the neonatal half-life elimination of clozapine. In this case, the infant was bottle fed, and was discharged from the hospital with his mother's family, awaiting surgery. Generalized neurodevelopmental delay was observed at the follow-up at 18 months. The mother was transferred to a mental institution 4 days after delivery to continue her treatment and clinical stabilization.

Three months after discharge, the same patient arrived in the Psychiatry Emergency Unit after alterations in her behavior. There was no evidence of a relapse in schizoaffective disorder. However, she was 6-weeks pregnant. In this new pregnancy (M3-2) she was hospitalized. Treatment was maintained with clozapine 200 mg/day throughout pregnancy, and sertraline 100 mg/day was added in gestational week 35 until postpartum. She gave birth to a girl in the 39th week of pregnancy by elective cesarean section. The Apgar-min 1/5 score was 9/10. The serum concentrations of clozapine/norclozapine in IMB and UCB were measured (Table 3). Figure 1 shows the neonatal half-life elimination of clozapine. Again, the neonate was bottle fed and was discharged from the hospital with his mother's family. No agranulocytosis or neurodevelopmental disorders were detected in the infant at the 6-month follow-up. The mother continued asymptomatic with the same treatment and she now attends the Mental Health Community Center.

## Discussion

In this report we present four cases of clozapine treatment during pregnancy focusing on the pharmacokinetics of clozapine and the maternal and neonatal outcomes. The placental passage of clozapine was partial in all four cases and the mean (SD) half-life value of clozapine in neonates who were exposed *in utero* was 92 (18) h. In the only case with maternal lactation we found no evidence of clozapine accumulation in the first 33 neonatal hours of life. One of the four pregnancies was complicated by diabetes mellitus. We did not observe any acute toxicological effects in three of the four neonates. The only neonate with respiratory problems was also exposed to maternal diazepam treatment. However, these results must be taken with caution being a case series with small sample size.

The current report indicates that placental passage of clozapine/norclozapine is partial during delivery (Table 2). These results contrast with those published previously by Barnas et al. (1994), who observed clear accumulation of clozapine in fetal serum. These differences in results could be explained by different maternal and fetal factors. The case by Barnas et al. (1994) was free of concomitant medication with no possible pharmacokinetic interaction. Our second case, treated also only with clozapine, showed a UCB/IMB ratio close to the mean ratio of the four cases. Another maternal factor to be taken into account is the fact that M1, M3-1, and M3-2 smoked during pregnancy. Pharmacokinetic studies have demonstrated lower clozapine /norclozapine concentrations in smokers compared to non-smokers (Lowe and Ackman, 2010). However, there was no information about smoking in Barnas' case report. Lastly, partial placental passage has been described in other atypical antipsychotics such as olanzapine, risperidone, and quetiapine as well as the first-generation antipsychotic haloperidol, suggesting a common pharmacokinetic behavior (Newport et al., 2007).

The elimination half-life of drugs has been related to adverse effects. Neonatal clozapine pharmacokinetics are thought to differ from those in adults (Jann et al., 1993) because cytochromes CYP450 3A4 and 1A2, which are responsible for demethylation of clozapine to norclozapine (desmethyl-clozapine) in adults, are present to a lesser extent in neonates (Myllynen et al., 2009). Furthermore, norclozapine is eliminated by glomerular filtration, which is known to be reduced in newborns (Smits et al., 2013). In our series the mean maternal, amniotic fluid and fetal concentrations of clozapine were higher than the concentrations of norclozapine. The clozapine/norclozapine concentrations were similar in amniotic fluid and fetal serum in our series. In contrast, in Barnas' case clozapine concentrations were similar in maternal serum and amniotic fluid, and there was a clear accumulation in fetal serum. In neonates we also observed higher concentrations of clozapine than norclozapine. The estimate of half-life elimination of clozapine by Moreno-Bruna et al. (2012) was 72 h. They found a delay in peristalsis that they hypothesized was related to the neonatal concentration of clozapine (200 ng/mL), which was indirectly estimated from the neonatal half-life elimination of clozapine. In our three neonates we found a mean half-life of clozapine of 92 h, with a mean plasma neonatal concentration of clozapine of 85(24) ng/mL in the first 24 h of life (Figure 1). This was not associated with any neonatal adverse effect. We must point out that our mean plasma neonatal concentration of clozapine was lower than that estimated by Moreno-Bruna et al. (2012). In the literature there are two cases of neonatal delayed peristalsis after maternal acute clozapine intoxication in late pregnancy in two women that were not receiving regular clozapine treatment (Kłys et al., 2007; Novikova et al., 2009). In the first case, the baby died after 3 h and the woman was saved. Post-mortem clozapine/norclozapine plasma levels were obtained. In the second, the baby developed abdominal distension on the first day of life, and the mother had a multisystemic failure and passed away after 3 days. Clozapine and its metabolites were detected in the urine of the mother at 3 days and the child at 6 days.

With regard to the lactation passage to clozapine, previous data (Barnas et al., 1994) suggested an accumulation in maternal milk. We were able to study lactation passage in only one mother (M2). The Food and Drug Administration (2005) suggests that the infant plasma concentration is probably the most direct measure of infant risk from a drug received from breast milk. In this sense, we observed that the infant/maternal plasma clozapine concentration ratio decreased close to 88% over time, from 0.557 (delivery day) to 0.065 (33 h post-delivery). In our case, the concentration of clozapine in the infant's plasma was ~6% of the corresponding maternal plasma drug concentration at 2nd day of life. Then, different to Barna's case our result did not suggest accumulation of clozapine and metabolite on the 2nd day of breastfeeding.

Finally, clozapine had no acute toxicological effects such as agranulocytosis or seizures, in the exposed neonates, regardless of exposure to other medications. However, one of the newborns was diagnosed with intrauterine growth retardation in the 28th gestational week, as well as left inguinal hernia and cryptorchidism at birth. Moreover, he presented with generalized neurodevelopmental delay and the consequent need for continuous intensive care. His mother had a history of past and current drug abuse (M3-1) during pregnancy. Importantly, the second baby born to this mother (M3-2) showed normal neurodevelopment. In this case, the baby was not exposed to maternal substance use other than tobacco. Regarding the long-term clinical outcome, signs and symptoms of attention deficit and hyperactivity disorder were observed (M1) after 6 years of follow-up.

## Ethics statement

This study was carried out in accordance with the recommendations of the Research and Ethical Committee (CEIC) of our Institution Hospital Clinic.

## Author contributions

All authors had full access to all of the data in the study and take responsibility for the integrity of the data and the accuracy of the data analysis. All authors: Case study concept and design. MI, GO, MT, DS, LG-E, and RM-S: Acquisition, analysis, or interpretation of data; MI, GO, and RM-S: Drafting of the manuscript; All authors: Critical revision of the manuscript for important intellectual content; MI, MT, and DS: Administrative, technical, or material support; All authors: Study supervision.

### Conflict of interest statement

The authors declare that the research was conducted in the absence of any commercial or financial relationships that could be construed as a potential conflict of interest.
